# A framework from point clouds to workpieces

**DOI:** 10.1186/s42492-022-00117-0

**Published:** 2022-08-23

**Authors:** Li-Yong Shen, Meng-Xing Wang, Hong-Yu Ma, Yi-Fei Feng, Chun-Ming Yuan

**Affiliations:** 1 School of Mathematical Sciences, University of Chinese Academy of Sciences, Beijing, 100049 China; 2 KLMM, Academy of Mathematics and Systems Sciences, University of Chinese Academy of Sciences, Beijing, 100049 China

**Keywords:** Computer numerical control, Mesh reconstruction, Mesh segmentation, Path planning, Scallop height

## Abstract

Combining computer-aided design and computer numerical control (CNC) with global technical connections have become interesting topics in the manufacturing industry. A framework was implemented that includes point clouds to workpieces and consists of a mesh generation from geometric data, optimal surface segmentation for CNC, and tool path planning with a certified scallop height. The latest methods were introduced into the mesh generation with implicit geometric regularization and total generalized variation. Once the mesh model was obtained, a fast and robust optimal surface segmentation method is provided by establishing a weighted graph and searching for the minimum spanning tree of the graph for extraordinary points. This method is easy to implement, and the number of segmented patches can be controlled while preserving the sharp features of the workpiece. Finally, a contour parallel tool-path with a confined scallop height is generated on each patch based on B-spline fitting. Experimental results show that the proposed framework is effective and robust.

## Introduction

Direct manufacturing from point clouds is a typical problem in reverse engineering and rapid prototyping. Some methods [1–5] can be used to directly generate the tool path from a point cloud for computer numerical control (CNC) machining.

These processes omit a global geometric construction, and the methods lose the essential geometric information (such as sharp features) during the machining process. For a point cloud, the local geometric information can be recovered using discrete techniques. However, this information is usually crude because the point cloud does not have a topological connectivity. It is therefore difficult to directly determine detailed geometric features from a point cloud. In this study, to address high-precision machining from geometric data, a framework is implemented from the point clouds to the workpieces, which can effectively determine and preserve sharp features.

Compared with point clouds, mesh models are widely used in computer-aided design and CNC owing to their simplicity and topological connectivity. The construction of a mesh from a point cloud is a frequently used process in geometric modeling. A mesh reconstruction algorithm primarily includes explicit and implicit reconstructions. Traditional methods include the ball-pivoting algorithm [6], crust algorithm [7], and Poisson surface reconstruction [8, 9]. With the development of deep learning, neural networks have also been used for mesh reconstruction. Explicit reconstruction methods based on neural networks [10, 11] work well for simple models but cannot deal with complex models. For complex models, implicit reconstruction methods exhibit a better performance. DeepSDF [12] uses a network to learn the signed distance of point to surface. To improve the geometric details, Gropp et al. [13] proposed implicit geometric regularization (IGR) by adding regular terms. These two implicit methods can be used to handle models with complex topologies and geometries.

The main purpose of mesh denoising is to remove mesh noise while retaining the sharp features of the object. Fleishman et al. [14] calculated the vertex positions through bilateral filtering. Rather than moving the vertex coordinates directly, Zheng et al. [15] applied a bilateral filter to normal vectors and then changed the vertex coordinates to obtain a better mesh than that achieved using a direct method. However, these two methods cannot preserve the sharp features. Although Zhang et al. [16] extended the total variation method to a mesh refinement, this approach tends to produce piecewise constant results. To overcome this drawback, Liu et al. [17] extended the total generalized variation (TGV) to a mesh, which is an approach called MeshTGV. As a key contribution, the authors constructed different facet operators and edge different operators and then used the variable-splitting and augmented Lagrange method (ALM) to solve the optimization problem. To obtain a mesh that can be used in CNC machining, in this study, IGR [13] is used to obtain a watertight model, and MeshTGV [17] is applied to denoise the mesh while preserving the sharp features.

Mesh segmentation is the process of dividing a complete model into several machinable surface patches and is an important step in a subtractive fabrication. This step can avoid collisions with the cutter during complex surface machining. Moreover, additional constraints can be applied to obtain better results, such as a fabrication direction constraint, the accessibility of the cutter, and setup and boundary constraints. Several methods have been proposed to address this problem. In addition to the discussions on additive fabrication [18–22], Mahdavi-Amiri et al. [23] introduced a novel carvable volume decomposition for efficient three-axis subtractive CNC machining of 3D freeform objects; however, their approach was for rough machining. Herholz et al. [24] proposed a segmentation method based on a graph cut. In addition, Zhao et al. [25] introduced the concept of accessibility when segmenting a mesh to make each segmented patch avoid collisions with the cutter. However, the theory behind these two methods is incomplete and an incorrect result may occur. Although the method in ref. [26] effectively avoids this problem, a series of optimization methods is used to ensure the accessibility of each patch, which complicates the method. The preservation of sharp features has always been an important research subject in CNC subtractive manufacturing. However, none of the above methods consider sharp features during segmentation, which makes it impossible to effectively the preserve sharp features in the subsequent tool path planning and CNC machining. To overcome this drawback, a novel segmentation algorithm is proposed that uses a minimum spanning tree (MST). This method is easy to implement and can effectively control the number of segmented patches while preserving the sharp features of the workpiece.

After segmentation, a machinable tool path satisfying the scallop height constraint must be generated. Tool path planning plays an important role in CNC manufacturing. A good tool path must satisfy the following requirements: a confined scallop height error, no self-intersection, and as few sharp corners as possible. Tool path planning methods can be briefly classified into two categories: topological and parametric. Methods based on a topological form mainly include direction parallel methods [27, 28], contour parallel methods [29–33], and space-filling curve methods [34–38]. Methods based on a parametric form mainly include iso-parametric methods [39, 40], iso-plane methods [41, 42], iso-scallop height methods [43–46], and vector field-based methods [47–51]. Among them, contour parallel methods are widely used in the industry owing to their simple topological properties. However, these contour parallel methods tend to ignore whether the scallop height of the path corner satisfies the constraint when generating the tool path. To ensure the machining quality and effectively preserve the sharp features, a contour parallel tool path generation method is provided that strictly satisfies the scallop height constraint.

**Fig. 1:**
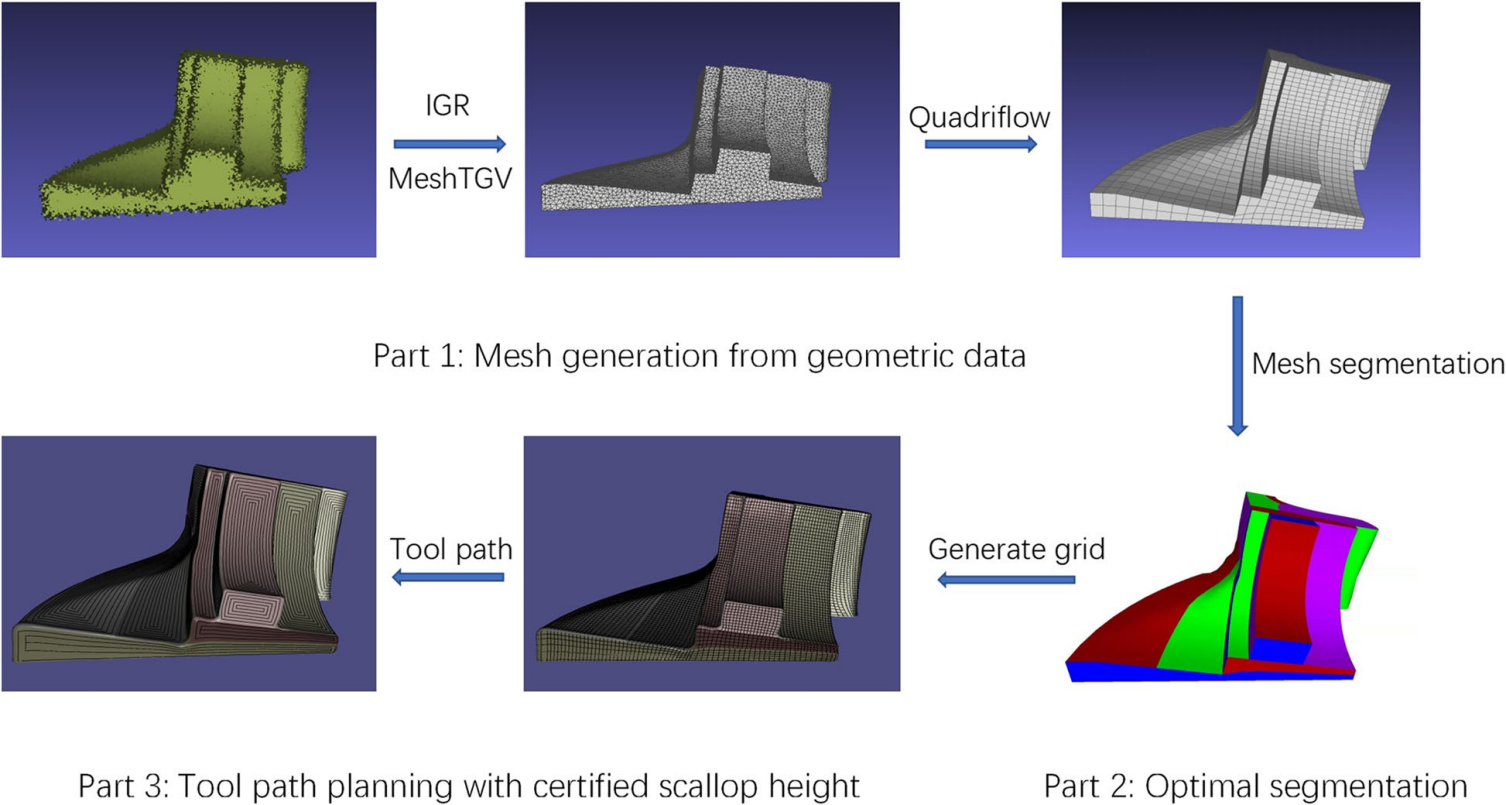
Pipeline of the total framework

In this study, a unified framework is proposed for integrating these components and successfully converting point clouds into machinable tool paths for the final CNC machining. The pipeline includes three main steps: mesh generation from geometric data, mesh segmentation for the CNC, and path planning under the scallop height constraint (Fig. 1).

The proposed framework for converting point clouds into workpieces has the following highlights: (1) A unified framework from geometric data to workpieces is achieved; (2) The proposed segmentation method is easy to implement and can control the number of segmented patches while preserving the sharp features of the workpiece; (3) The generated contour parallel tool path of the proposed method strictly satisfies the scallop height constraint.

The remainder of this paper is organized as follows. The following section describes the methods used, which are divided into design, mesh generation, optimal surface segmentation method, and tool path planning with certified scallop height. The results are presented in Results section. The advantages and problems of the new approach are then discussed in Discussion section. Finally, some concluding remarks are provided in Conclusions section.

## Methods

### Design

In this study, to address high-precision machining from geometric data, a framework is implemented from the point clouds to the workpieces. The goals of the framework are as follows: (1) obtain the essential geometric information (such as sharp features) of the point clouds, (2) preserve the sharp features during machining, and (3) generate a machinable tool path.

To accomplish the above objectives, the proposed framework can be divided into three parts: mesh generation from geometric data, optimal surface segmentation for CNC, and tool-path planning with the certified scallop height. Further details of these objectives are provided in the following sections.

### Mesh generation from geometric data

**Fig. 2:**
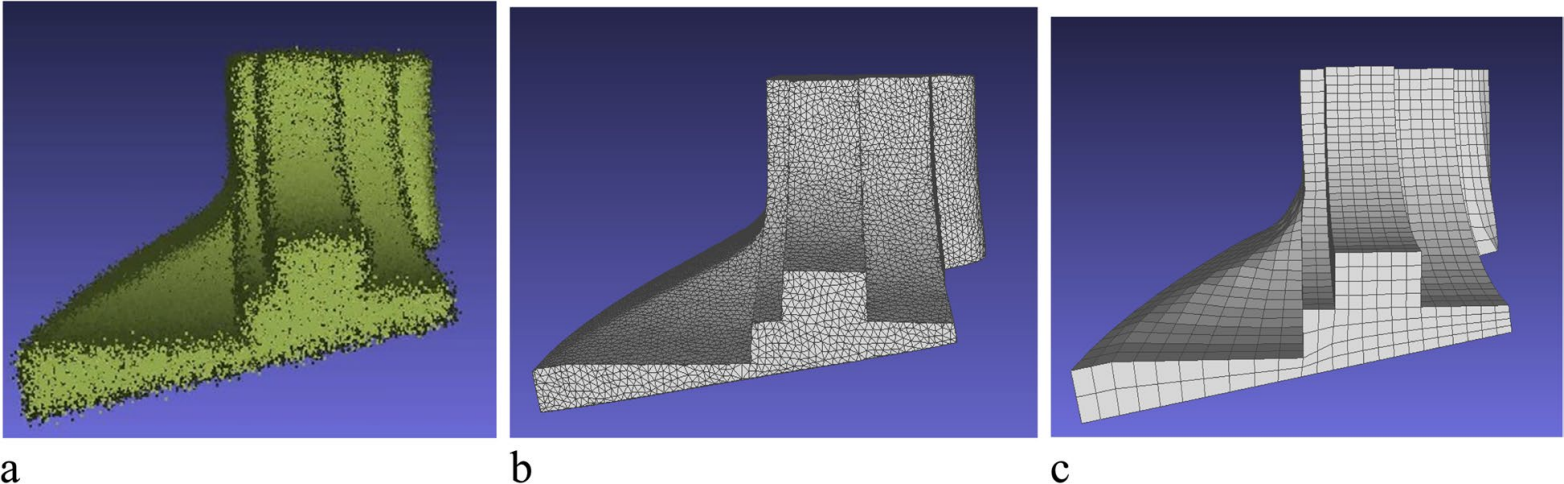
From point cloud to mesh. **a** Point cloud; **b** Denoised mesh; **c** Final mesh

In this subsection, the methods used to generate a mesh model are briefly introduced. The point cloud is converted into a mesh using IGR [13], and the mesh is then denoised by applying MeshTGV [17]. The results show that IGR can produce a better mesh than the other mesh reconstruction methods, and MeshTGV can preserve sharp features without artifacts. To facilitate subsequent operations, a triangular mesh is converted into a quadrilateral mesh using Quadriflow [52]. An example of this is shown in Fig. 2.

#### IGR

Given an input point cloud *χ* = {***x***_***i***_}_*i* ∈ *I*_ ⊂ *ℝ*^3^, with or without normal data, $\mathcal{N}={\left\{{n}_i\right\}}_{i\in I}\subset {\mathbb{R}}^3$. IGR learns a signed distance function $f:{R}^{\mathbb{3}}\times {R}^m\to R$ using a multilayer perceptron. The loss function is defined as follows:

1 $$\ell \left(\theta \right)={\ell}_{\chi}\left(\theta \right)+\lambda {\mathbb{E}}_x{\left(\left\Vert {\nabla}_{\boldsymbol{x}}f\left(\boldsymbol{x},\theta \right)\right\Vert -1\right)}^2$$

where *λ* > 0, and ‖∙‖ is *L*_2_ − *norm*.

2 $${\ell}_{\chi}\left(\theta \right)=\frac{1}{\left|I\right|}\sum\limits_{i\in I}\left(\left|f\left(\boldsymbol{x},\theta \right)\right|+\tau \left(\left\Vert {\nabla}_{\boldsymbol{x}}f\left(\boldsymbol{x},\theta \right)\right\Vert -{n}_i\right)\right)$$

The architecture is similar with DeepSDF and is easy to implement.

#### MeshTGV

Here, MeshTGV is used to refine a noisy mesh obtained from IGR by removing the noise [17]. With MeshTGV, some difference operators are constructed on the edges and facets of the mesh and a normal filter is applied to remove the noise while preserving the sharp features. The normal filter process is converted into a non-differentiable optimization problem. Variable splitting and ALM are introduced to solve this problem and find a smoothed normal field. Subsequently, the vertices are updated to generate the final mesh model using the scheme proposed by Zhang et al. [53].

#### Quadriflow

QuadriFlow is used to convert a triangular mesh into a quadrilateral mesh. QuadriFlow was developed from Instant Meshes [54] and has certain constraints added. Specifically, the regularity constraint is enforced by minimizing the cost network flow, and the consistent orientation constraint is then successively enforced. According to these constraints, one can re-optimize the position field and extract the quadrilateral mesh from the position field.

### Optimal surface segmentation for CNC

To effectively preserve the sharp features of the workpiece during processing, the cutter should follow a path parallel to the sharp features rather than intersecting them. The mesh obtained in the last subsection is therefore segmented into several patches in such a way that the sharp features are located on the boundaries of each patch. A contour parallel tool path can then be generated on each patch to effectively preserve the sharp features. In this subsection, the proposed mesh segmentation method, which uses a MST, is introduced. Herein, the following new concept is also introduced:

Definition 1 *A quadrilateral mesh patch E*_*i*_ *is called a sharp height field (SHF) if it satisfies the following conditions:*

1. *E*_*i*_ *is quadrilateral.*
2. *There are no extraordinary points in E*_*i*_.
3. *The sharp features are located on the boundaries of* *E*_*i*_.

Starting from the generated quadrilateral mesh *F* described in the previous subsection, the goal here is to segment the mesh into SHFs and control the number of SHFs using techniques applied in graph theory:

3 $${E}_i\subseteq F,{\bigcup}_i{E}_i=F,{E}_i\cap {E}_j=\varnothing$$

where *E*_*i*_ is the SHF segmented from the given mesh.

In Definition 1, conditions 1 and 2 ensure that the SHF can be fitted using a B-spline surface. After segmentation, a tool path that satisfies the scallop height constraint on each SHF must be generated. During this process, the first- and second-order differential information of the workpiece surface must be known; however, it is difficult to obtain accurate differential information for the surface represented by a mesh. A B-spline surface is therefore used to fit the SHF after segmentation, for which conditions 1 and 2 were considered. Condition 3 preserves the sharp features of the workpiece during the processing.

**Fig. 3:**
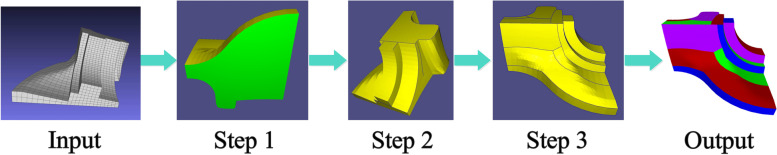
Pipeline of the segmentation method

The segmentation method consists of three steps (Fig. 3): first, the K-means clustering algorithm is used to remove the workpiece setup base of the mesh; second, a weighted graph of the mesh is constructed based on the location of extraordinary points and sharp features. The MST of the graph is then obtained to determine the optimal connection of the extraordinary points; third, the mesh is divided into several SHFs by determining the clipping edges according to a specific priority.

#### Removal of workpiece setup base

In real CNC machining, one can machine the base first and then use it for the machining setup to deal with the remaining parts. A setup base for the mesh must therefore first be chosen and removed. Meanwhile, removing the base can simplify the connection of extraordinary points (Eps, an interior vertex in a quadrilateral mesh which is shared by other than four faces), thus simplifying the subsequent segmentation process.

**Fig. 4:**
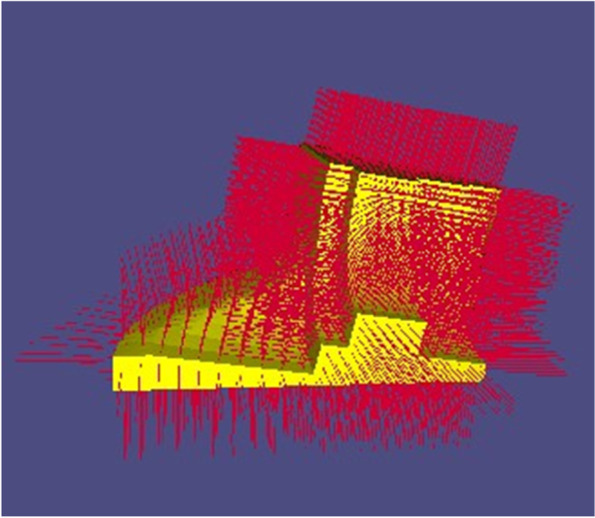
Outer normal vectors (red lines) on the mesh

In general, the base should be as flat and large (in terms of area) as possible. Here, the K-means clustering algorithm is used to select a suitable base. First, the outer normal vector of each facet on the mesh is obtained, as shown in Fig. 4.

**Fig. 5:**
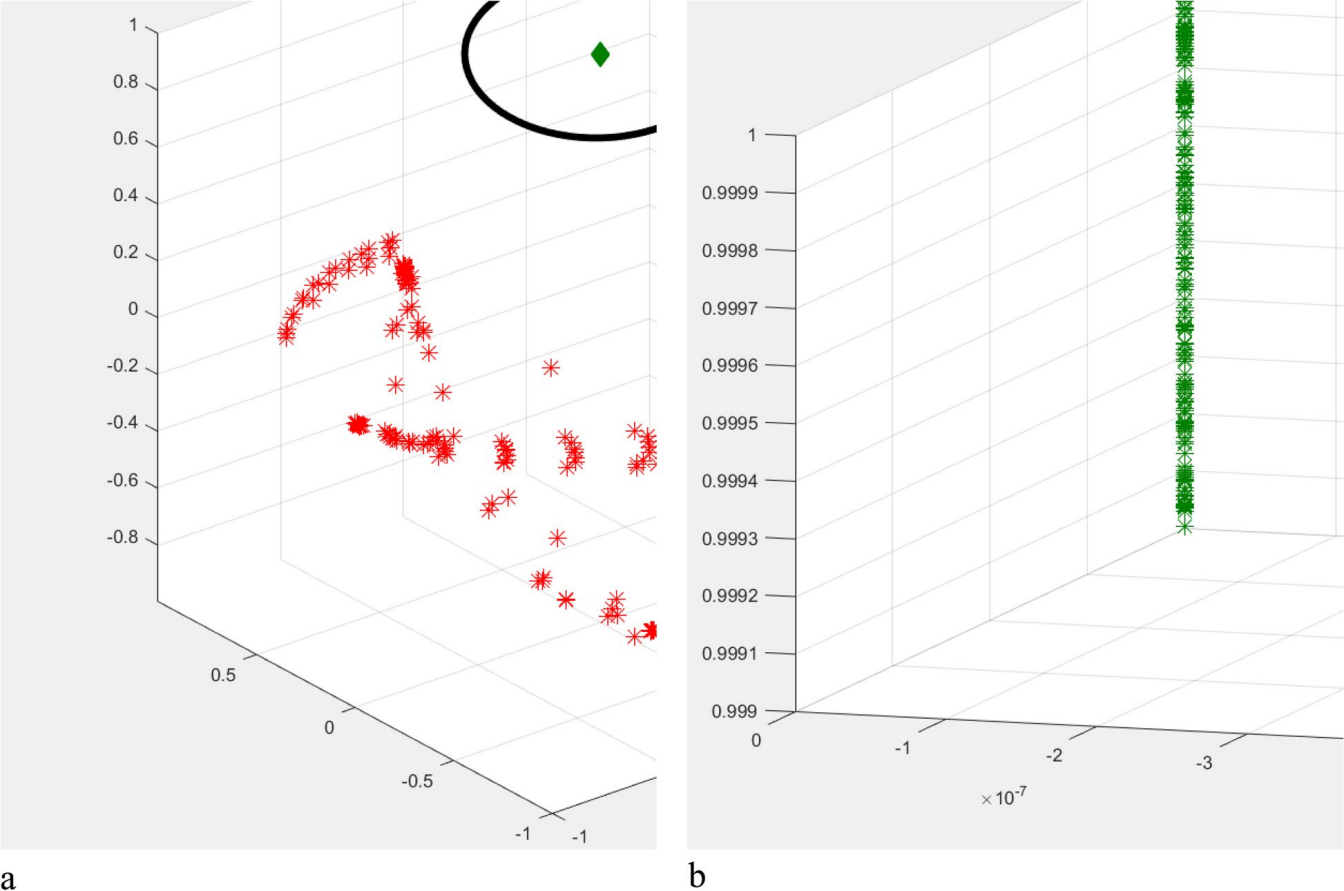
Data points representing normal vectors. **a** Optimal dataset is shown in green; **b** Local enlarged portion of the optimal set

**Fig. 6:**
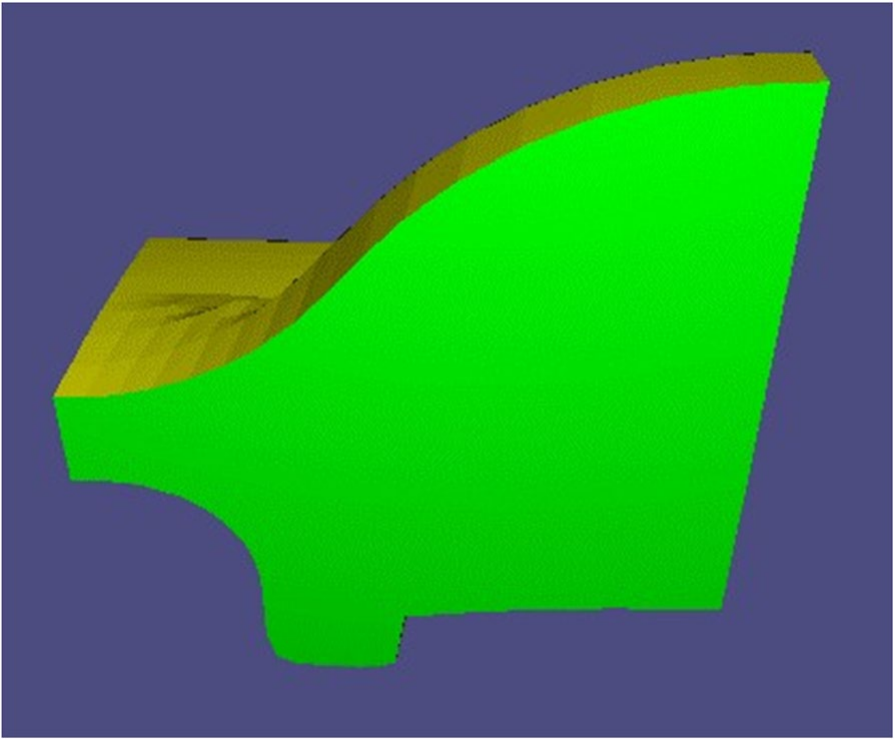
Setup base of Fandisk model

Subsequently, each normal vector is treated as a three-dimensional data point, and the K-means clustering algorithm is used to classify such data points. The following objective function is then provided:

$$\upepsilon =\upmu \cdot S+\upgamma \cdot \Gamma$$

where *S* and Γ are the similarity and quantity of each dataset, respectively, and μ and γ are two weighting coefficients. Here, let μ = 0.7 and γ = 0.3. This objective function is used to obtain the optimal set of data points, as indicated by the black circle in Fig. 5. The optimal set of data points is mapped onto the workpiece, which is the setup base chosen in this study (with a one-to-one correspondence between faces and their normal vectors). As shown in Fig. 6, the green part represents the setup base, which is removed from the mesh.

#### MST of extraordinary points

**Fig. 7:**
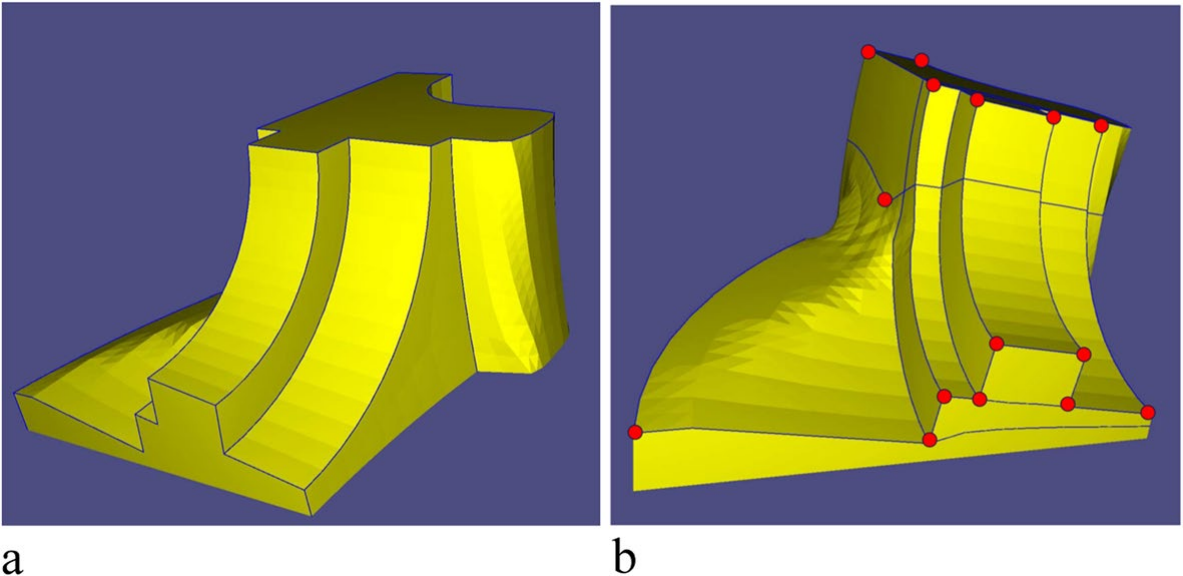
Information on Fandisk model. **a** Sharp features; **b** Edges between EPs (red dots)

After removing the base, conditions 2 and 3 in Definition 1 are considered. For condition 3, which edges are sharp features must first be determined. Here, the angle between the normal vectors is used to determine whether the edge is a sharp feature: An edge with two adjacent quadrilateral facets is judged to be a sharp feature if the angle between the corresponding normal vectors of the two facets is greater than a given degree *θ* (in this study, let *θ* = 50^∘^). In Fig. 7a, the blue lines represent sharp features of the Fandisk model.

For condition 2, the EPs should be located at the boundaries of each SHF; therefore, all connection edges between extraordinary points are considered, as shown in Fig. 7b. In this way, the initial segmentation problem can be regarded as a paper-cut problem with EPs and sharp features on the clipping edges. Which mesh edges are clipping edges must be determined such that Eq. (3) is satisfied. Owing to condition 2, some connection edges of the EPs belong to the clipping edges. The connection relations of the EPs directly determine the number of SHFs; hence, the connections of the EPs must be controlled and connected together.

**Fig. 8:**
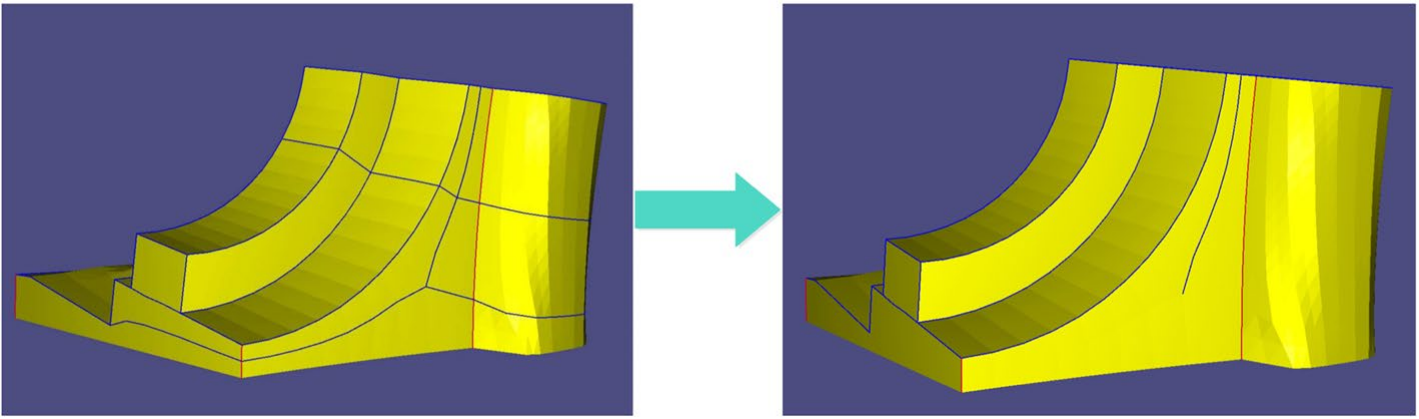
Removing redundant connection edges of EPs

First, the connected weighted graphs of the EPs are established. In Fig. 7b, all connection edges naturally form a connected graph. According to condition 3, all sharp features belong to the clipping edges, and thus the sharp features that do not belong to the connection edge of the EPs (called priori sharp edges) are in a sense equivalent to the boundary of the mesh, as shown by the red lines in Fig. 8. Hence, the unnecessary connection edges of the EPs that intersect the priori sharp edges should be removed. Subsequently, the weighted graph on the mesh is defined.

**Fig. 9:**
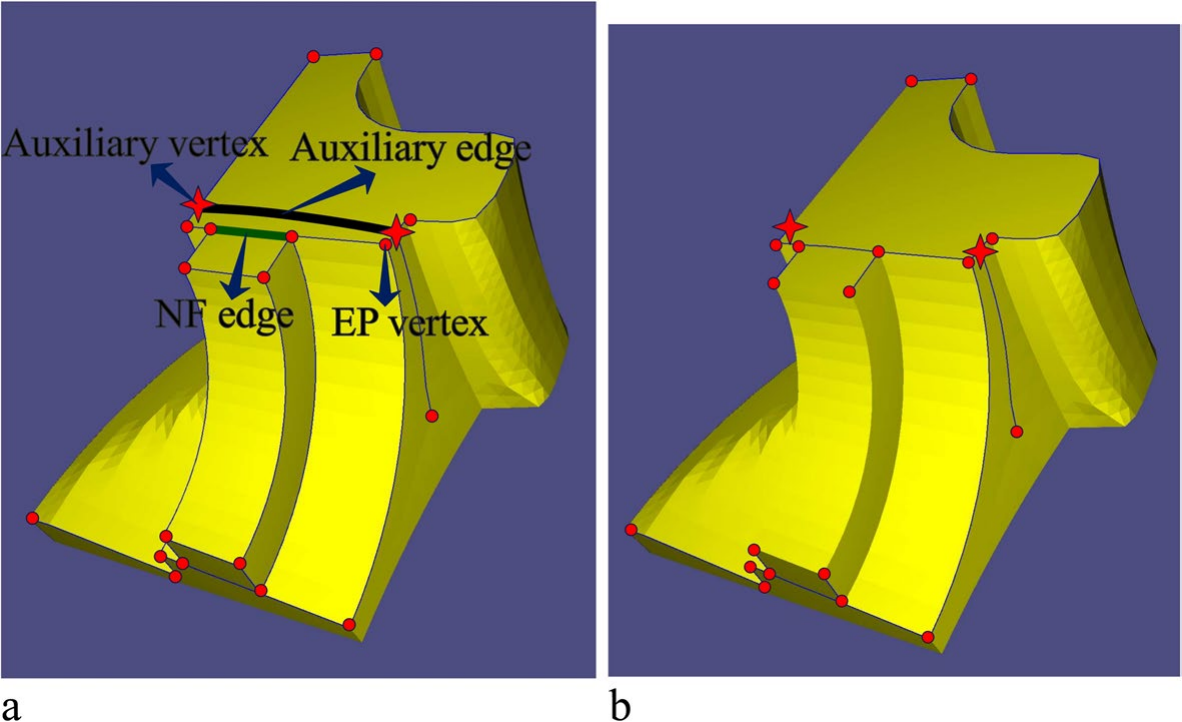
Information of connected graph and its MST. **a** Weighted graph of the mesh; **b** MST of connected graph

Two types of vertices are given in the weighted graph, i.e., an EP vertex (red dots in Fig. 9) and an auxiliary vertex (red stars in Fig. 9). The EP vertex corresponds to the extraordinary points, and the auxiliary vertex is formed by the intersection of the two connection edges of the EPs. The remaining connection edges between the EPs form the edges of the weighted graph.

**Fig. 10:**
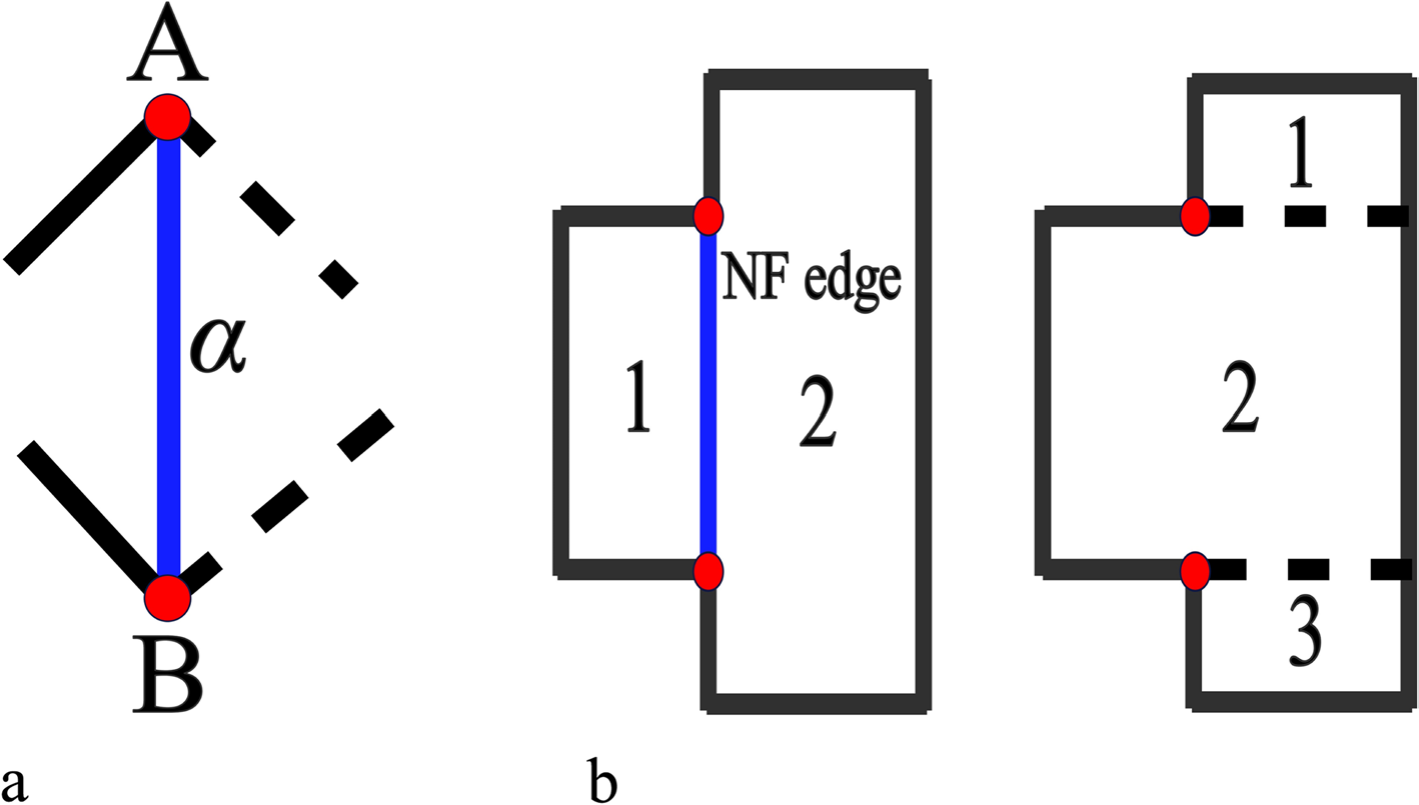
Information on NF edge. **a** Definition of NF edges; **b** Comparison of various connections

The weight of each edge is then obtained. There are three types of edges: auxiliary, non-full degree of freedom (NF), and standard. The connecting edge of the two auxiliary points is called the auxiliary edge (black line in Fig. 9a). Choosing this type of edge as the clipping edge often increases the number of SHFs in the segmentation problem. Thus, the weight of the auxiliary edge will be set to a large number. The definition of NF edge is as follows: When the two endpoints of edge *α* are EPs A and B, the four edges adjacent to edge *α* at the EPs have at least one edge that does not belong to the weighted graph (the black dotted line in Fig. 10a), and *α* is then called an NF edge. Choosing an NF edge as a clipping edge often decreases the number of SHFs in the segmentation problem. As shown in Fig. 10b, if an NF edge is chosen as a clipping edge, the number of SHFs can be reduced to two, unlike with other clipping edges. Thus, smaller weights are given to this type of edge. In addition to the two types of edges mentioned above, the remaining edges in the graph are called a standard edge. The weights of these edges are as follows:

4 $${\displaystyle \begin{array}{c}{weight}_{standard}={\sum}_{i\in \Xi}{SC}_i\cdot {length}_i\\ {}\begin{array}{c}{weight}_{NF}={\sum}_{i\in \Xi} NC\cdot {SC}_i\cdot {length}_i\\ {}\begin{array}{c}{weight}_{auxiliary}={\sum}_{i\in \Xi} AC\cdot {SC}_i\cdot {length}_i\\ {}{SC}_i=\left\{\begin{array}{c}0.1,i\in sharp\ feature\\ {}1, otherwise\end{array}\ \right.\end{array}\end{array}\end{array}}$$

where Ξ is the set of boundaries of the quadrilateral facets that belong to the edge of a connected graph, *length*_*i*_ is the length of the boundary of a quadrilateral facet, *SC*_*i*_ is the coefficient of the sharp features, *NC* is the *NF* edge coefficient (set to 0.1), and *AC* is the auxiliary edge coefficient (set to 1000).

Because some redundant connection edges of the EPs are removed, there may be multiple connected weighted graphs on the mesh (for example, a single extraordinary point can be regarded as a degenerate weighted graph). The MST for each graph is determined to obtain the optimal connection relation between the EPs. Herein, the Kruskal greedy algorithm [55] is adopted to obtain the MST, as shown in Fig. 9b.

#### Remaining clipping edges

After obtaining the MST, the clipping edges on the mesh must be determined to obtain the SHFs. The clipping edges are set sequentially according to the following priorities: (1) MST of each weighted graph, (2) sharp feature edges and mesh boundaries, (3) NF edges, (4) extended edges of the sharp features, and (5) redundant connected edges for EPs.

**Fig. 11:**
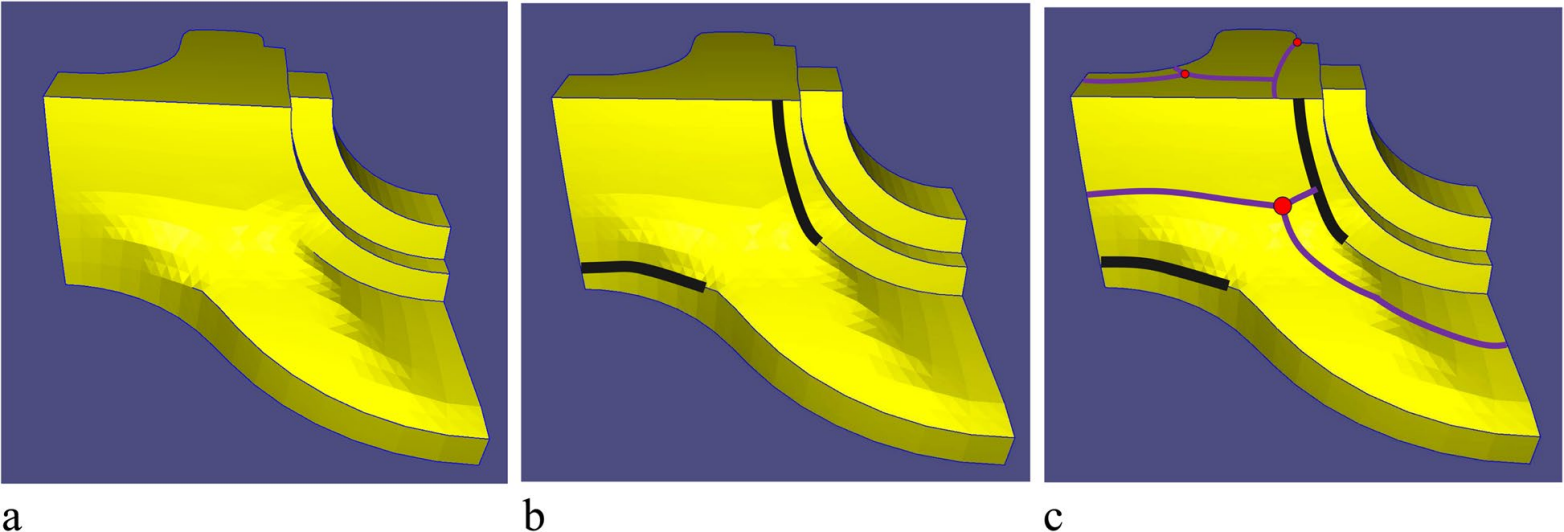
Information on clipping edges. **a** Priorities 1, 2, and 3; **b** Priority 4 (black curve); **c** Priority 5 (purple curve)

These edges are set as clipping edges in order. Priorities 1–3 have been defined. As for priority 4, when the first three items are set as clipping edges, the entire mesh is insufficient to divide into several patches, as shown in Fig. 11a. Therefore, sharp features that do not intersect other clipping edges must be selected and extended forward until they meet other clipping edges (black curves in Fig. 11b), which is priority 4. The mesh can therefore be segmented into several patches.

**Fig. 12:**
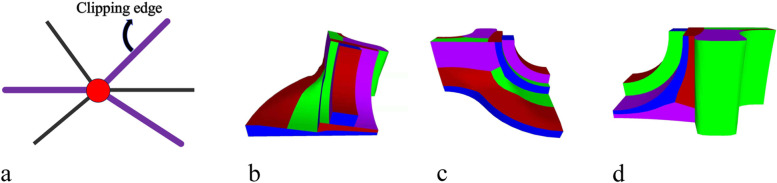
Results of Fandisk model. **a** Clipping edges of Eps; **b** Front view; **c** Left view; **d** Right view

For priority 5, the redundant (non-clipping edge) connecting edges of the EPs must be set as clipping edges to satisfy condition 1 in Definition 1. A clipping edge is thus set for every other connecting edge of the EPs, as shown in Fig. 12a. They are extended forward until they meet the other clipping edges (purple curves in Fig. 11c). Subsequently, the entire optimal segmentation algorithm is completed. Figures 12b-d show the results of the Fandisk model.

### Tool path planning with certified scallop height

In this subsection, a tool path generation method for each SHF is presented. To ensure the machining quality and effectively preserve the sharp features, a contour parallel tool path that strictly satisfies the scallop height constraint is chosen. During this process, although the first- and second-order differential information of the workpiece surface must be known, it is difficult to obtain accurate differential information for surfaces represented by a mesh. Therefore, a B-spline surface is used to fit the SHFs after segmentation prior to the tool-path generation.

Given a set of initial control meshes (the SHFs are used as the initial control mesh) and a target triangle mesh (generated denoised mesh described in MeshTGV section), the surface should be fit using a B-spline surface. Simultaneously, the result must ensure that *C*^0^ is continuous at the boundary of the SHFs. In this study, uniform bi-cubic B-spline surfaces are used to fit the mesh because the topological structure of each SHF is simple.

**Fig. 13:**
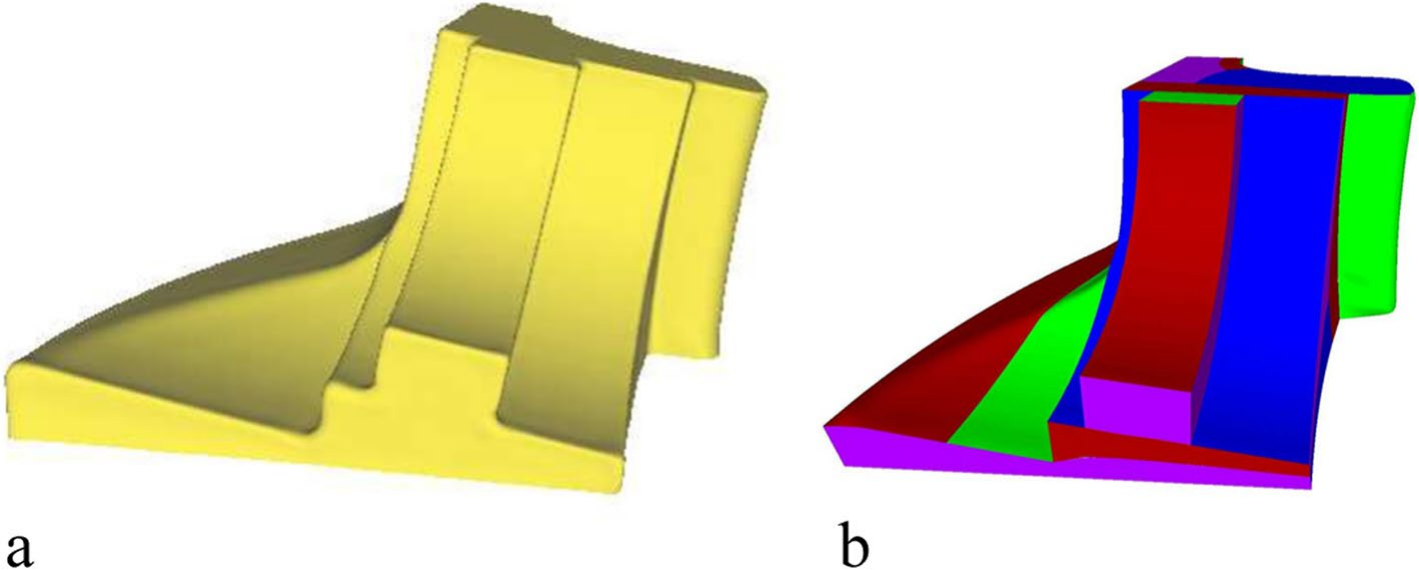
Fitted spline surface of Fandisk model. **a** Fitted spline surface; **b** Fitted SHFs

Random points *P* are obtained from the triangle mesh during every iteration, and the error between the point cloud and the B-spline surface should be minimized. First, the as-rigid-as-possible method proposed in ref. [56] is used to parameterize the point cloud *P*. Instead of using the Cartesian distance, the feature-sensitive metric in ref. [57] is applied. This metric is defined in the position and normal spaces in R^6^, which is more sensitive to surface changes. A local quadratic approximation of the squared distance method (SDM), is then used, as proposed in ref. [58]. During each iteration, the following energy function is optimized:

$$f=\frac{1}{2}\sum\limits_{k=1}^n{e}_k+\alpha {F}_1$$

where *e*_*k*_ is the SDM error term, and *F*_1_ is the thin planar energy. Because each part term is quadratic, the optimization problem can be transformed into the solution of a system of linear equations. The optimization problem is then solved iteratively until the error between the triangle mesh and spline surface becomes less than the threshold. Finally, the fitted B-spline surfaces are obtained, as shown in Fig. 13.

**Fig. 14:**
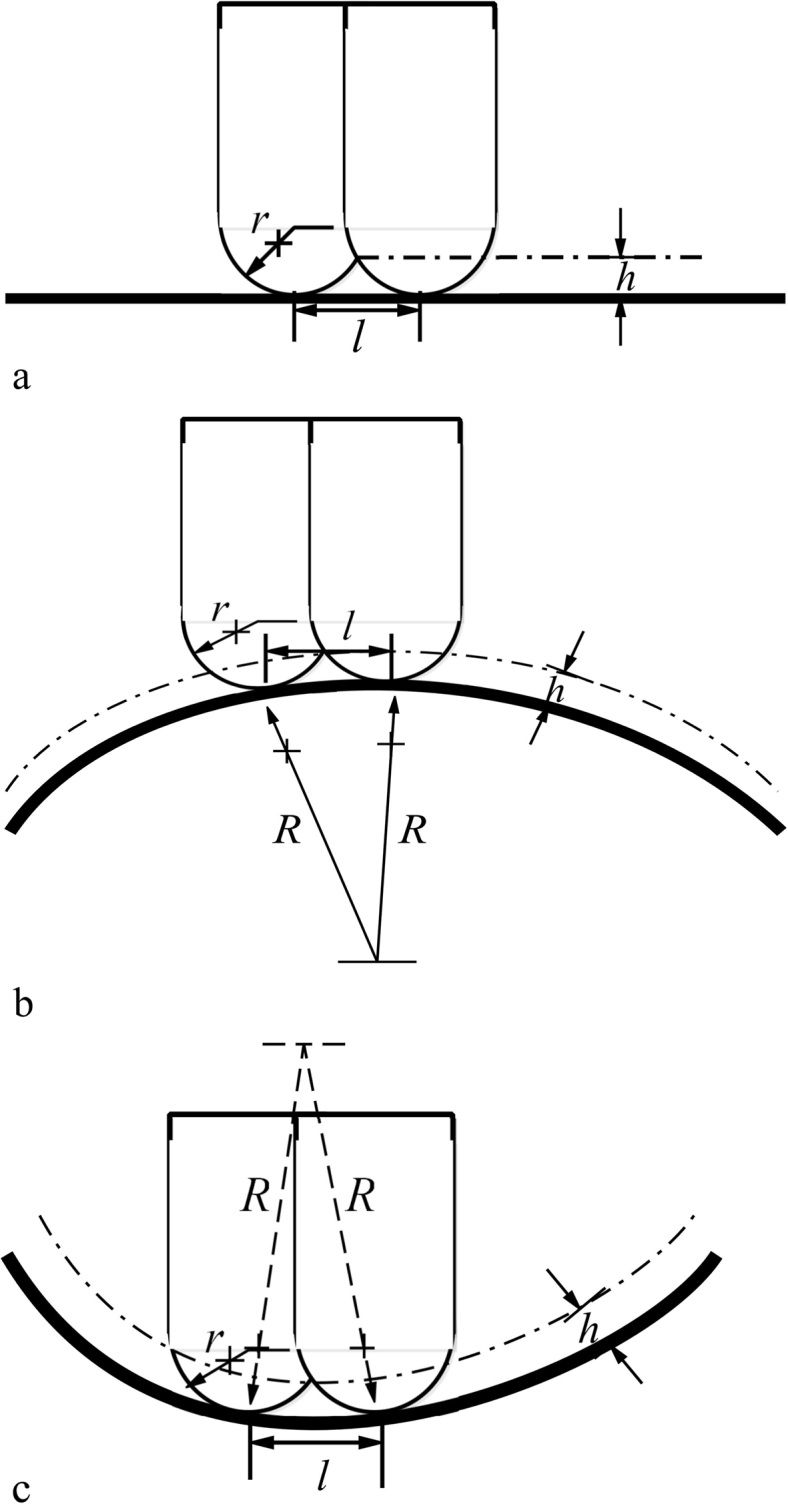
Path intervals on three different surfaces. **a** Path interval on flat plane; **b** Path interval on a convex surface; **c** Path interval on concave surface

Subsequently, a tool path is generated on each fitting surface. First, a grid that satisfies the scallop height constraint on the rectangular parameter plane is provided. Before generating a grid, the path interval must be calculated according to the scallop height constraint. Scallops are created on the machined surface when the cutter moves along the tool path. The distance between paths is the cutter contact (CC) path interval, as shown in Fig. 14. The calculation of the path interval is associated with the convexity of the surface. In this study, a ball-end cutter is used to machine the surface, and the CC path interval then depends on the local radius of normal curvature *R* of the surface, the feedrate direction, the radius *r* of the cutter, and the scallop height *h* remaining on the surface. In general, the scallop height *h* is much smaller than radius *r* of the cutter; hence, the calculation of the path interval can be simplified. Through machining using a ball-end cutter, the tool path interval for different surfaces can be estimated from the following equations [59]:

$${l}_{flat}=2\sqrt{2 rh},{l}_{convex}=\sqrt{\frac{8 hrR}{R+r}},{l}_{concave}=\sqrt{\frac{8 hrR}{R-r}}$$

When the path interval *l*_*i*_ is obtained, it can be mapped back to the parameter domain using the following two formulas, and the distance of adjacent CC points in the parameter plane can be obtained [37]:

5 $$\Delta s=\frac{\pm {l}_i\left(F\frac{ds}{du}+G\frac{dt}{du}\right)}{\sqrt{EG-{F}^2}\sqrt{E{\left(\frac{ds}{du}\right)}^2+2F\frac{ds dt}{du du}+G{\left(\frac{dt}{du}\right)}^2}}$$

6 $$\Delta t=\frac{\mp {l}_i\left(E\frac{ds}{du}+F\frac{dt}{du}\right)}{\sqrt{EG-{F}^2}\sqrt{E{\left(\frac{ds}{du}\right)}^2+2F\frac{ds dt}{du du}+G{\left(\frac{dt}{du}\right)}^2}}$$

Here, *E*, *F*, and *G* are the first fundamental parameters of the surface, and *u* is the parameter of the current tool-path curve.

Subsequently, with the scallop height constraint satisfied, Algorithm 1 is used to generate the grid, as shown below:

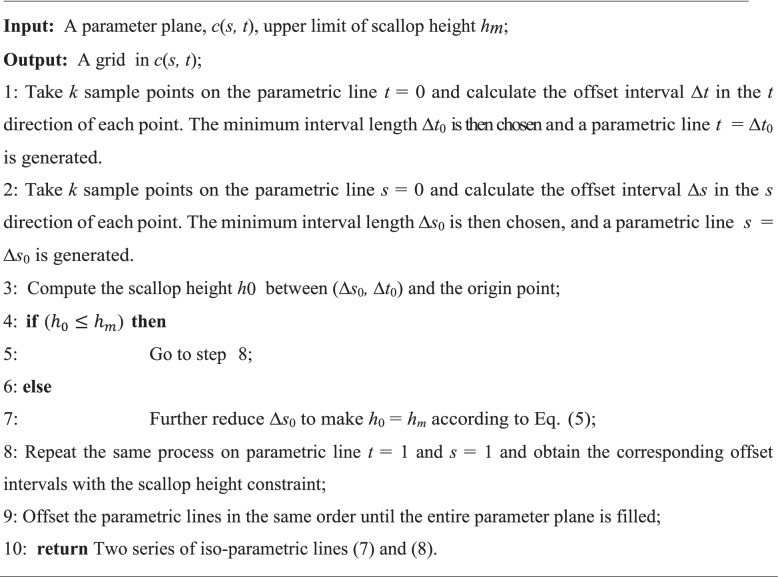



**Fig. 15:**
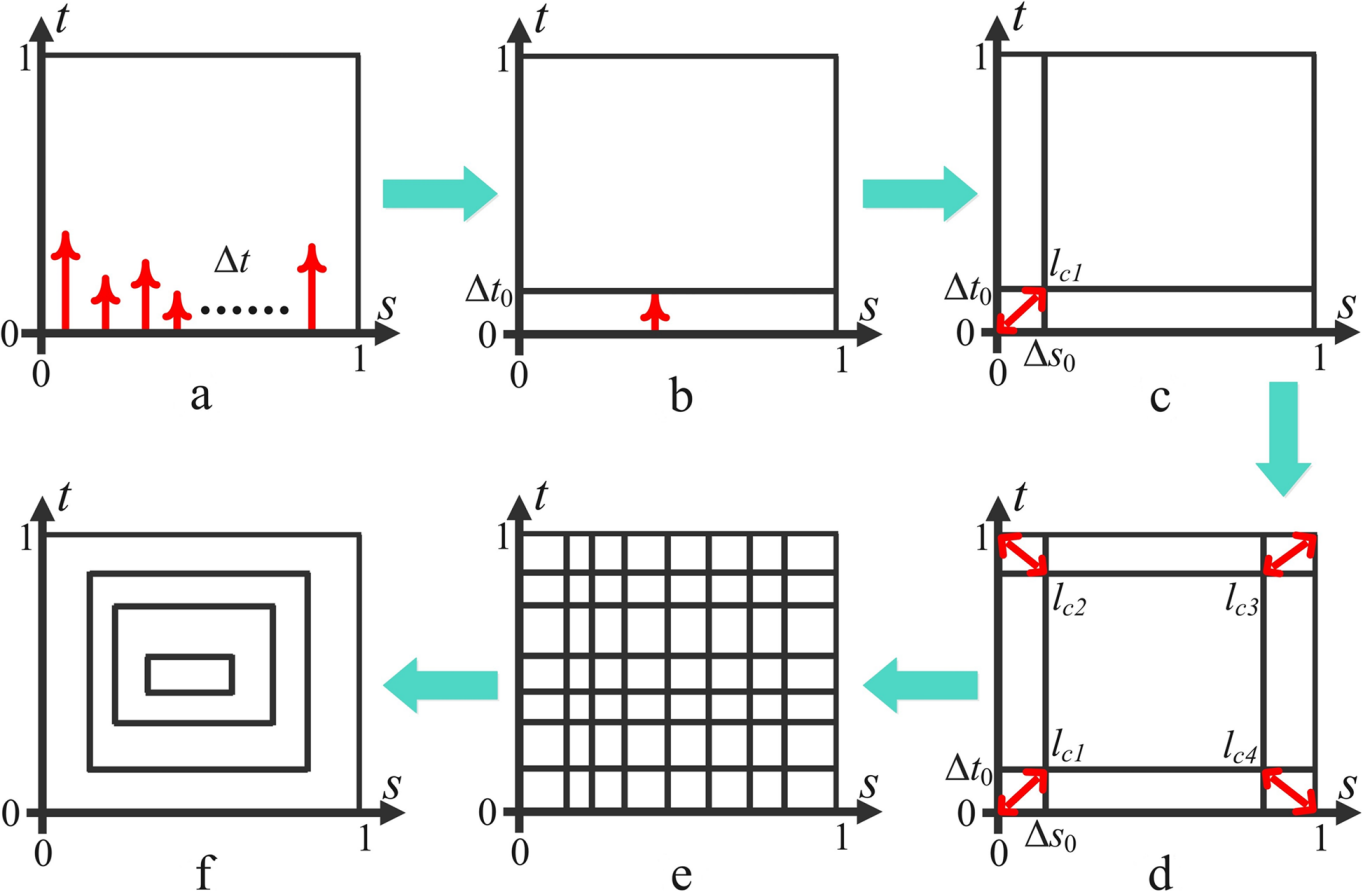
Pipeline of the tool path generation method. **a** The offset interval of each sample point; **b** The minimum interval is selected to obtain the parametric line; **c**, **d** Adjust the intersection point to satisfy the scallop height constraint; **e** Offset the parametric lines until the entire parameter plane is filled; **f** Generate the contour tool paths

More specifically, *k* sample points are taken from the lowest boundary of the perimeter plane, that is, the parametric line *t* = 0. For each sample point, the offset interval ∆*t* is calculated in the *t* direction with a given upper limit of the scallop height *hm*, as shown in Fig. 15a. Subsequently, the minimum is selected and defined as ∆*t*_0_ to obtain the parametric line *t* = ∆*t*_0_, as shown in Fig. 15b. The same process is repeated on the parametric line *s* = 0, and the minimum interval ∆*s*_0_ is obtained. Next, whether the scallop height between intersection point *lc*_1_ of the two parametric lines and the origin point is less than *h*_*m*_ is considered. If the scallop height constraint is not satisfied, ∆*s*_0_ is further reduced to make the scallop height between the two points equal to *hm* according to Eq. (5), as shown in Fig. 15c. Next, the above process is repeated on parametric lines *t* = 1 and *s* = 1, and the corresponding offset intervals are obtained with the scallop height constraint (Fig. 15d). Finally, the parametric lines are offset in the same order until the entire parameter plane is filled (Fig. 15e).

In this manner, any two adjacent points on the grid satisfy the scallop height constraint. As a result, two series of iso-parametric lines are defined as follows:

7 $$\left\{t={t}_0=\Delta {t}_0,t={t}_1=\sum\limits_{i=0}^1\Delta {t}_i,\cdots, t={t}_N=\sum\limits_{i=0}^N\Delta {t}_i\right\}$$

8 $$\left\{s={s}_0=\Delta {s}_0,s={s}_1=\sum\limits_{i=0}^1\Delta {s}_i,\cdots, s={s}_M=\sum\limits_{i=0}^M\Delta {s}_i\right\}$$

After obtaining the grid, a cluster of contours can be naturally generated as the tool path of the surface (for example, the first contour is composed of four parametric lines: *t* = *t*_0_, *t* = *t*_*N*_, *s* = *s*_0_, and *s* = *s*_*M*_), as shown in Fig. 15f. Thus, a tool path for the surface is generated.

**Fig. 16:**
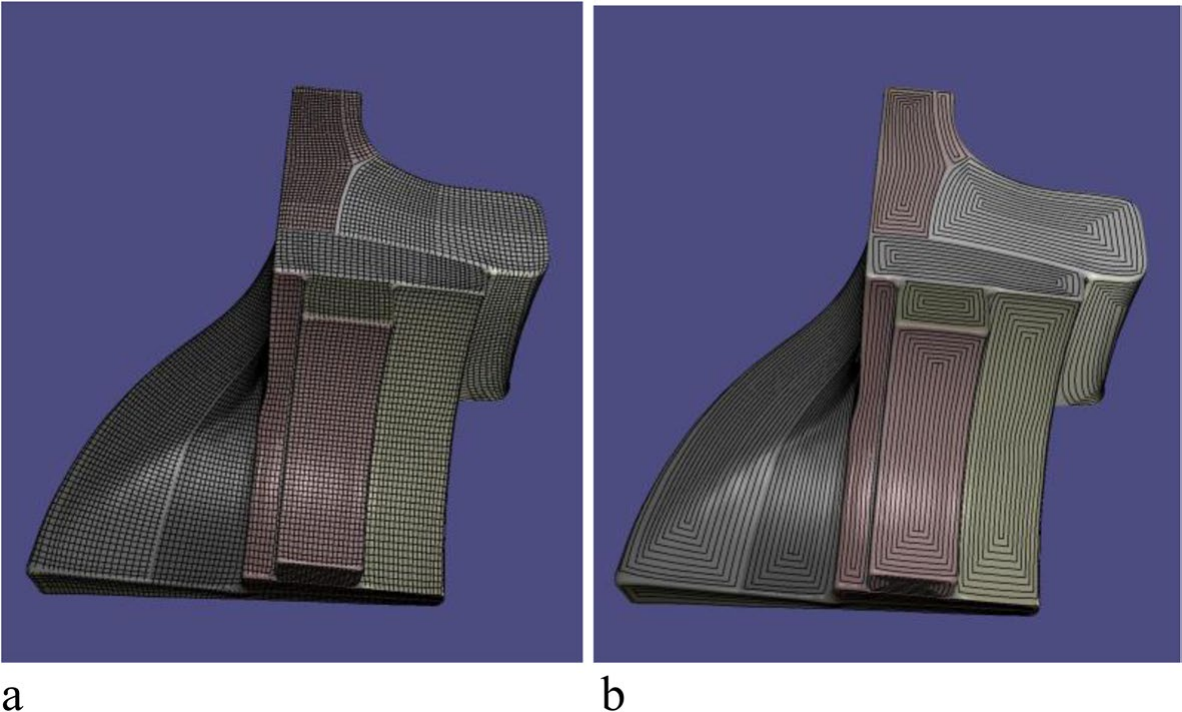
Whole contour parallel tool paths of Fandisk model. **a** Grids; **b** Contour tool paths

Finally, a grid with a scallop height constraint and corresponding contour parallel tool path for every SHF is generated, and the entire tool path planning algorithm is completed. Figure 16 shows the final results.

## Results

In this section, the process of converting a point cloud into a workpiece is described. The entire framework contains three parts: mesh generation from geometric data, optimal surface segmentation for CNC, and tool path planning with the certified scallop height. The results demonstrate the validity of this framework.

The proposed surface segmentation and tool path generation methods were implemented in C++ on a desktop computer with 16 GB of RAM, a 3.50-GHz Intel®Core™ i5-6600k CPU, and Windows 10. The official implementations of IGR, MeshTGV, and Quadriflow are used. For the given test point cloud, the framework can generate a contour parallel tool path that strictly satisfies the scallop height constraint and effectively preserves the sharp features. The scallop height is set to 0.16 mm.

### Mesh generation from geometric data

**Fig. 17:**
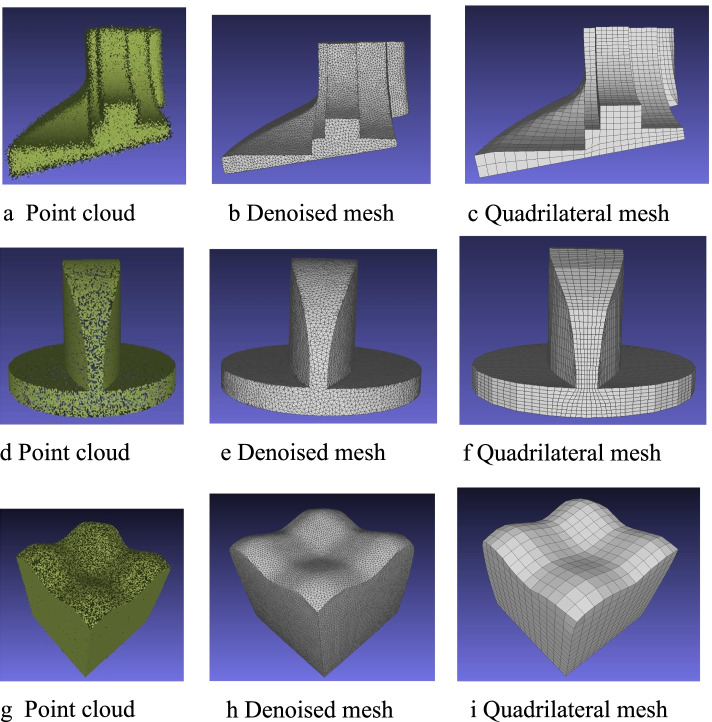
From point cloud to mesh: **a**-**c** Fandisk models; **d**-**f** Spheredisk models; **g**-**i** Surfcube models

The reconstruction results are shown in Fig. 17. For each model, the point cloud is sampled, and a triangle mesh is successfully generated while preserving the sharp features using IGR and MeshTGV. In addition, the triangle mesh is converted into a quadrilateral mesh using QuadriFlow.

### Optimal surface segmentation for CNC

**Fig. 18:**
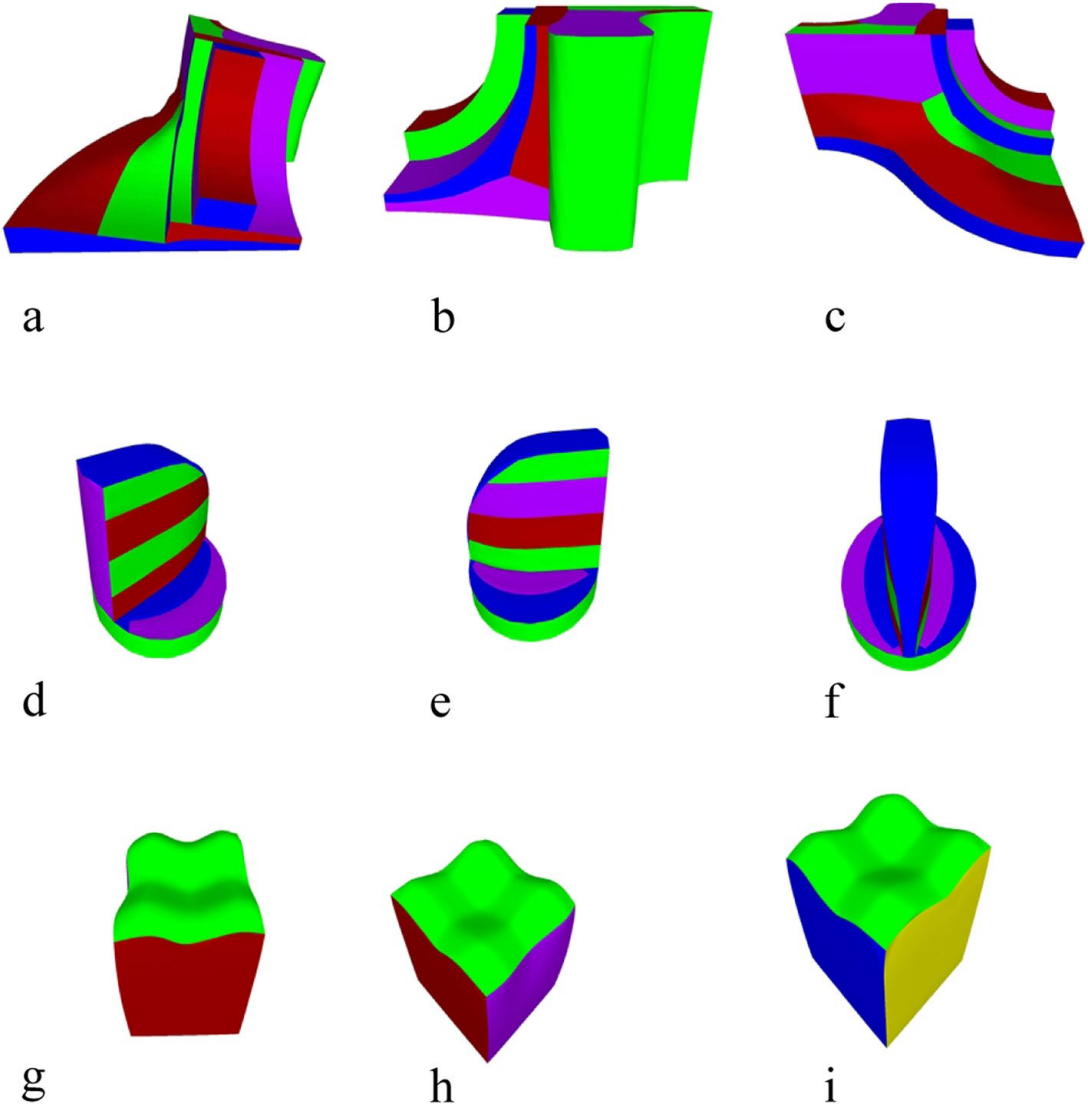
Mesh segmentation: **a**-**c** Fandisk model; **d**-**f** Spheredisk model; **g**-**i** Surfcube model

The segmentation results are shown in Fig. 18, and the proposed algorithm was tested on a quadrilateral mesh obtained in the previous section. First, a cluster algorithm is used to remove the workpiece setup base of the mesh and an MST is constructed based on the EPs. Finally, the mesh is divided into several SHFs by determining the clipping edges according to a specific priority. The results show that the proposed surface segmentation method is suitable for subtractive manufacturing and can control the number of segmented patches.

### Tool path planning with certified scallop height

**Fig. 19:**
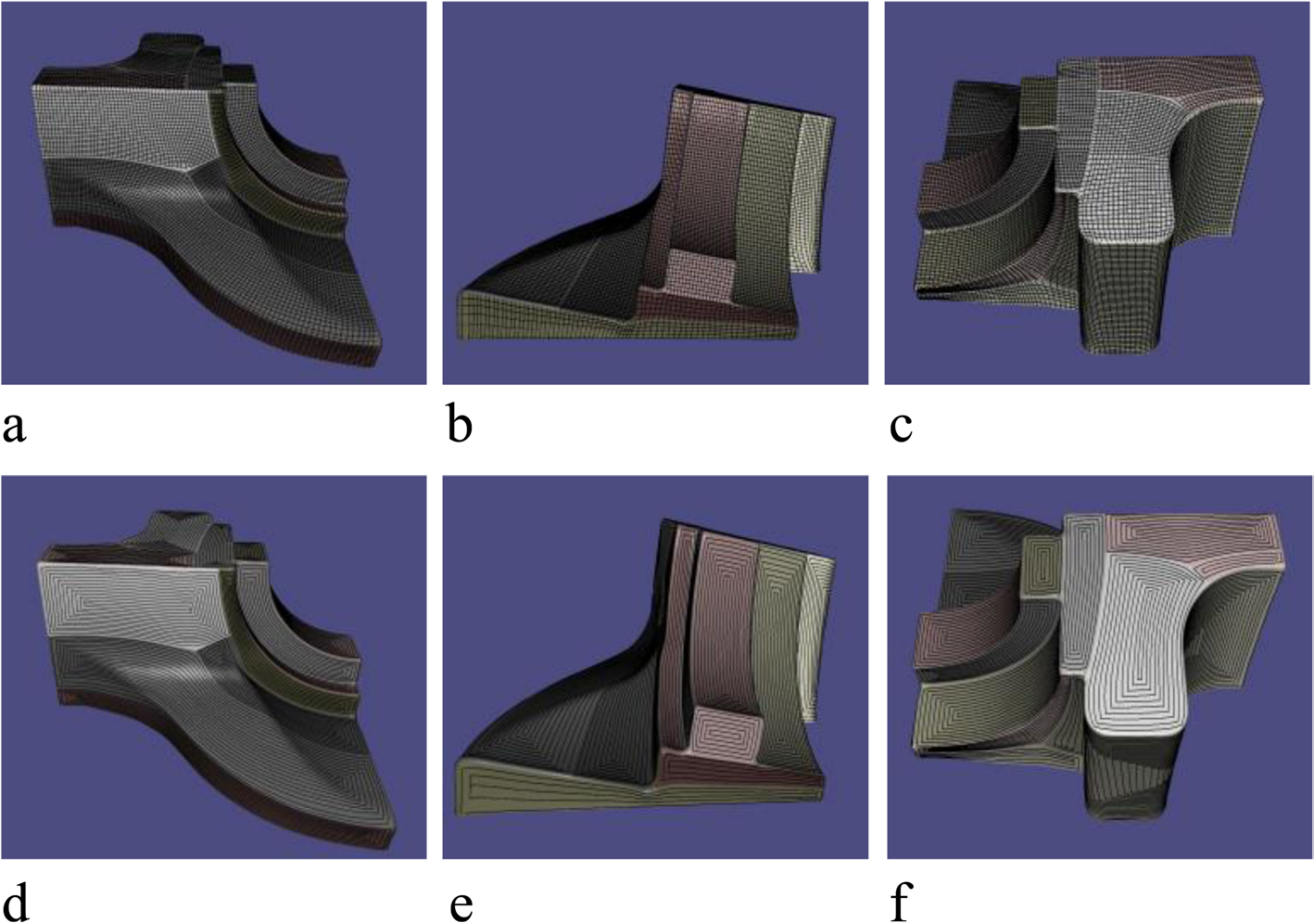
Tool path of Fandisk model: **a**-**c** are model’s grid; **d**-**f** are corresponding tool path

**Fig. 20:**
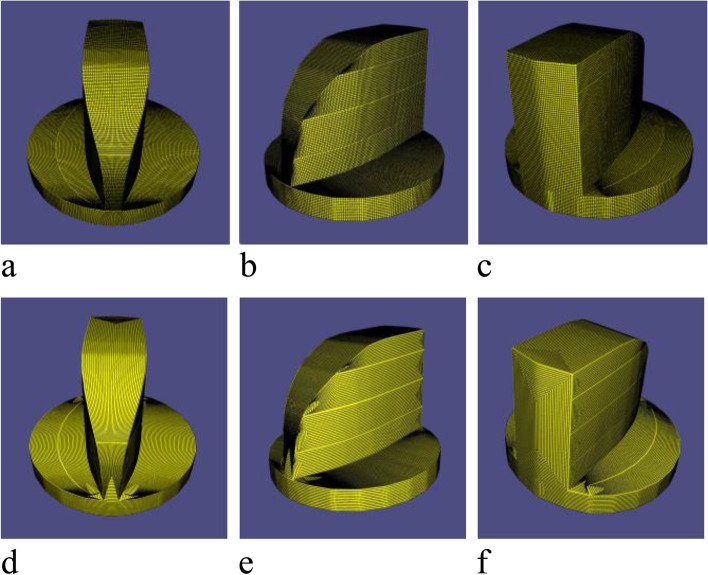
Tool path of Spheredisk model: **a**-**c** are model’s grid; **d**-**f** are corresponding tool path

**Fig. 21:**
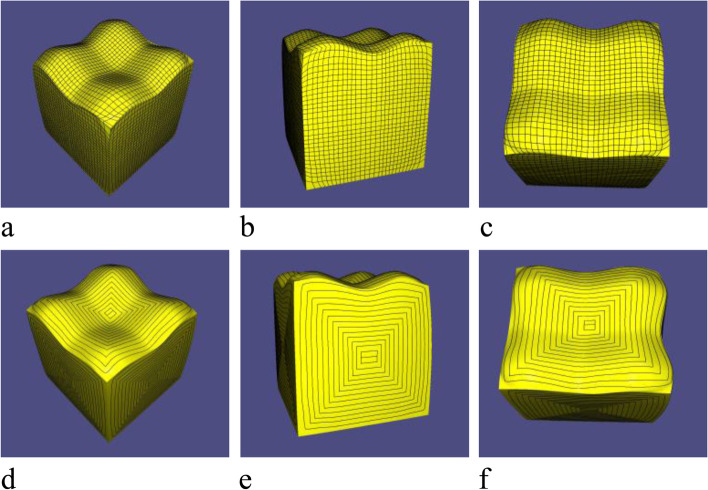
Tool path of Surfcube model: **a**-**c** are model’s grid; **d**-**f** are corresponding tool path

In this subsection, the contour parallel tool path results generated through the proposed method are presented. First, a grid on every SHF is generated, and a cluster of contours is then naturally obtained as the tool paths of the surface. The results show that the generated tool paths strictly satisfy the scallop height constraint and effectively preserve sharp features. Figures 19, 20 and 21 show the results.

## Discussion

With the increasingly extensive application of point clouds in various industries, their manufacturing has become an interesting topic of interest in the manufacturing process. The proposed framework can directly generate a machining tool path with a confined scallop height. The proposed optimal segmentation method is the first technique that effectively preserves the sharp features of the workpieces during the machining.

Meanwhile, the proposed segmentation method can divide a mesh into SHFs and control the number of SHFs using graph theory techniques. However, proof that the proposed method can minimize the number of SHFS was not provided, which will be an area of future study.

Note that with the above tool path generation method, when the cutter processes the workpiece along contour parallel lines, it can be ensured that the machining scallop height is below the given value, *hm*. Although this tool-path planning algorithm is conservative (resulting in dense paths), it can ensure the quality in CNC machining.

## Conclusions

In this study, a unified framework from point clouds to workpieces was proposed. An optimal segmentation method for subtractive manufacturing was also presented. To the best of the authors’ knowledge, this method is the first technique that effectively preserves the sharp features of workpieces during machining. In addition, the proposed method is easy to implement and can control the number of segmented patches. The contour parallel tool path is then generated using a parametric grid, which strictly satisfies the scallop height constraint. In summary, the proposed framework converts point-cloud data into a machinable tool path.

In a future study, the segmentation method will be improved to minimize the number of SHFs. Focus will be on the iso-scallop height tool path generation method on each SHF to effectively shorten the length of the tool path.

## Data Availability

The IGR application is available at https://github.com/amosgropp/IGR. The MeshTGV application is available at https://github.com/LabZhengLiu/MeshTGV and Quadriflow is available at https://github.com/hjwdzh/QuadriFlow.

## Abbreviations

CNC: Computer numerical control
IGR: Implicit geometric regularization
TGV: Total generalized variation
ALM: Augmented Lagrange method
SHF: Sharp height field
MST: Minimum spanning tree
EP: Extraordinary point
NF edge: Non-full degree of freedom edge
SDM: Squared distance method
CC: Cutter contact
